# Pathway-focused PCR array profiling of CAL-27 cell with over-expressed ZNF750

**DOI:** 10.18632/oncotarget.23075

**Published:** 2017-12-09

**Authors:** Li Pan, Hongli Yang, Wenqiang Tang, Cong Xu, Shuangfeng Chen, Zhen Meng, Keyi Li, Haiying Chen

**Affiliations:** ^1^ Oral Maxillofacial Head-Neck Key Laboratory of Medical Biology, and Central Laboratory of Liaocheng People's Hospital, Liaocheng, Shandong 252000, China; 2 Department of Oral and Maxillofacial Surgery, Liaocheng People's Hospital, Liaocheng, Shandong 252000, China

**Keywords:** Zinc-finger protein 750 (ZNF750), oral squamous cell carcinoma (OSCC), signal transduction, PCR array

## Abstract

Zinc-finger protein 750 (ZNF750) is the potential anti-cancer gene in oral squamous cell carcinoma (OSCC). The present study was to investigate the expression changes of ZNF750 in OSCC tissue and to reveal the induction of altered mRNA expression profiles caused by over-expressed ZNF750 in CAL-27 cell. The expression level of ZNF750 in tissue specimens from OSCC patients was detected by immunohistochemistry. Gene expression profiling was performed using Human Signal Transduction PathwayFinder RT^2^ Profiler™ PCR Array. The expression changes of 84 key genes representing 10 signal transduction pathways in human following over-expressed ZNF750 in CAL-27 cell was examined. The expression of ZNF750 protein was reduced in OSCC tissues. The R^2^ PCR Array analysis revealed that 39 of the 84 examined genes that changed at least a two-fold between control and ZNF750 groups. These genes related to oxidative stress, WNT, JAK/STAT, TGFβ, NF-kappaB (NFκB), p53, Notch, Hedgehog, PPAR and Hypoxia signaling. ZNF750 could inhibit the candidate genes ATF4, SQSTM1, HMOX1, CCND1, TNF-alpha, TNFSF10 and FOSL1 but activate CDKN1A and EMP1. Our studies suggest that ZNF750 can regulate signaling pathways that related to proliferation, cell cycle, inflammation and oxidative stress in CAL-27 cell.

## INTRODUCTION

Oral cancer is an aggressive and lethal disease with no significant improvements in the overall survival in the last decades [1]. Most oral cancers are squamous cell carcinomas that predominantly develop from cell hyperplasias and dysplasias [2]. The mortality and morbidity rates of oral squamous cell carcinoma (OSCC) remain high [3]. It is reported that Zinc-finger protein 750 (ZNF750) is normally expressed in keratinocytes [4], and is key factor to turn on the terminal epidermal differentiation gene program. ZNF750 not only repress epidermal progenitor genes but also induce differentiation genes [5]. Therefore, the abnormal expression of ZNF750 in oral keratinocyte may lead to imbalance of cell proliferation and development to malignant tumor. As for ZNF750 is a newly found lineage-specific tumor suppressor in squamous cell carcinoma [6], there is still a great deal of work to be done to identify the effect of ZNF750 on OSCC.

Earlier study reported that ZNF750 is the only known gene residing in focal deletion in head and neck squamous cancers [7]. The loss of ZNF750 is associated with impaired differentiation and failure to fully repress the proliferative genetic program [8]. Therefore, ZNF750 deletion may initiate OSCC cellular signaling for development. Cancer is a typical disease exhibited abnormality cell signal transduction [9, 10]. Cellular signaling forms a complex network of gene interactions involving multiple signal transduction pathways [11]. Each pathway ultimately increases or decreases the expression of its target genes resulting in alteration of cellular processes. Changes in target gene expression suggest signaling pathway activation or inhibition [12, 13]. As we mentioned above, ZNF750 is a crucial squamous cancers-specific suppressor, and our previous studies have found its differentiation-induction and cell invasion and migration inhibition effects in OSCC cell line CAL-27 cell [14]. In the present study, we aim to explore the ZNF750 expression level in OSCC tissue, and focus on investigation the gene expression profile upon over-expressed ZNF750 in CAL-27 cell, to evaluate the role of ZNF750 in its regulation.

## RESULTS

### The expression of ZNF750 protein in OSCC tissues

**Figure 1:**
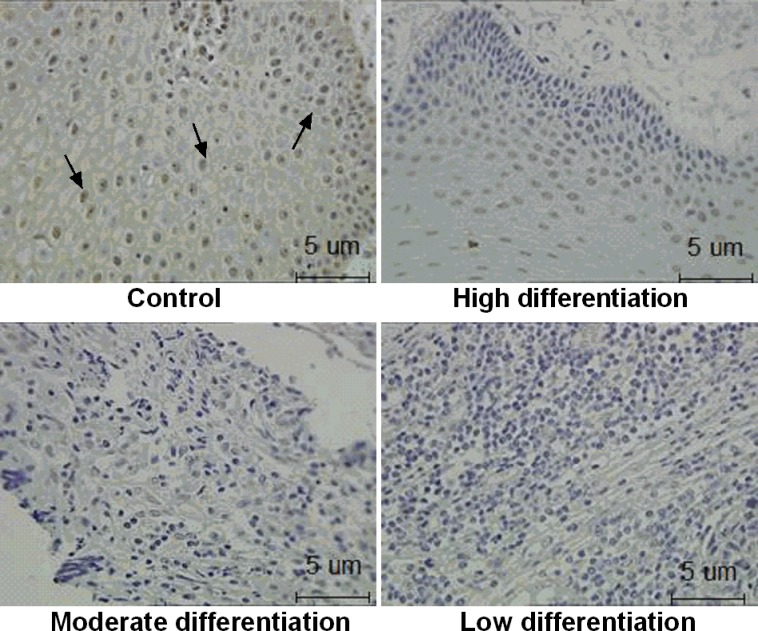
The expression of ZNF750 in OSCC tissues The ZNF750 protein expression was investigated by immunohistochemistry. Positive brown staining for ZNF750 is indicated by the arrow. Control: The adjacent normal tissues. High, moderate, and low differentiation: Represent for tumor differentiation. The ZNF750 protein expression was weak or nearly not detectable in OSCC tissues than adjacent normal tissues.

The present study observed the expression of ZNF750 protein in OSCC tumors and its adjacent normal tissues. There was many positive brown staining for ZNF750 in control groups. Compared to adjacent normal tissues, there was no detectable ZNF750 expression in all OSCC tissues with moderately and low differentiation (*p* < 0.01), only 4 out of 43 detected weak expression of ZNF750 in high differentiation OSCC tissues (Figure 1, Table 1).

**Table 1:** ZNF750 protein expression in OSCC tissue

Group	Positive (number)	Negative (number)	Total (number)
Normal	12	0	12
High differentiation	4	39	43
Moderately and low differentiation	0	42	42
Total	16	81	97

*X*^2^= 53.863, *p* < 0.01.

### Gene expression profiles of signal transduction in CAL-27 cell under ZNF750 over-expression

**Figure 2:**
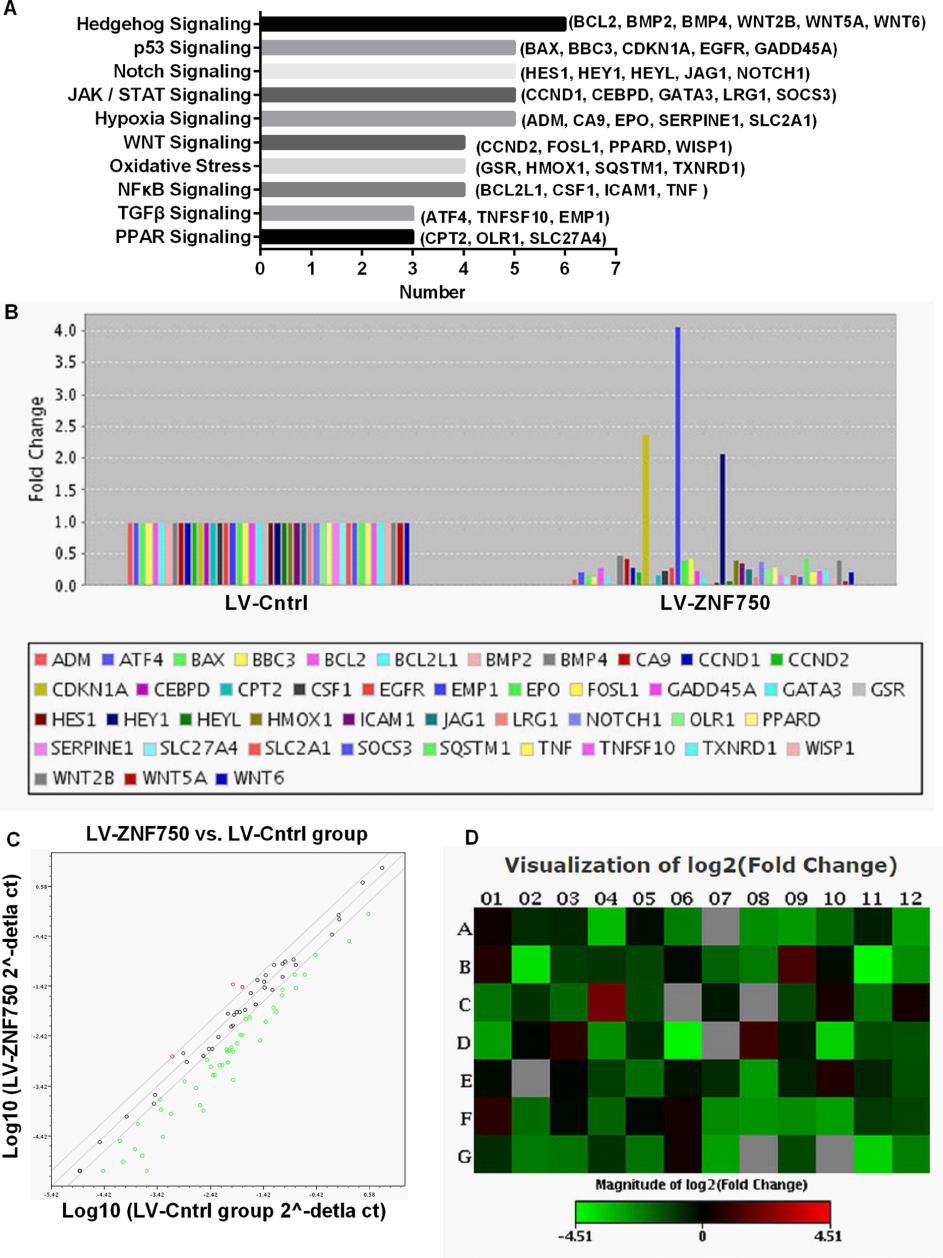
Effects of ZNF750 on gene expression profiles of signal transduction in CAL-27 cell The expression change of the 84 genes in ZNF750 groups and LV-Cntrl groups was performed by real-time PCR based array analysis. The expression levels in LV-Cntrl were set as 1. (**A**) The bar diagram is the clustering of differential expression genes in 10 signal transduction. (**B**) Genes that changed at least a two-fold differential expression in LV-ZNF750 group against LV-Cntrl group. (**C**) The red and green dot stands for up-regulated and down-regulated genes respectively. (**D**) The red and green color represent for increasing or decreasing genes in LV-ZNF750 group against LV-Cntrl group respectively showed in the heat map.

The differential mRNA expression levels of the 84 genes involved in the common signaling pathway were assessed. The bar diagram indicate the clustering of differential expression genes in 10 signal transduction pathway, these 10 signal pathway were related to cell proliferation, cell apoptosis, cell cycle, inflammation, hypoxia and oxidative stress. There were more changed genes in Hedgehog, p53, Notch, JAK/STAT and hypoxia signaling pathway (Figure 2A). The present study found that 44 out of the 84 examined genes that changed at least a two-fold differential expression in LV-ZNF750 group against LV-Cntrl group, three genes were up-regulated and 41 genes were down-regulated (Figure 2B). For scatter plot, the central line indicates unchanged gene expression. The genes expression changes are beyond the line boundary were showed in red dot for up-regulated genes, or green dot for down-regulated genes (Figure 2C). A heat map provides a graphical representation of fold expression in LV-ZNF750 group against LV-Cntrl group, red and green color represent increasing or decreasing genes respectively (Figure 2D). These differential expression genes related to cell proliferation, cell cycle, inflammation, hypoxia and oxidative stress.

### Validation for candidate gene

**Figure 3:**
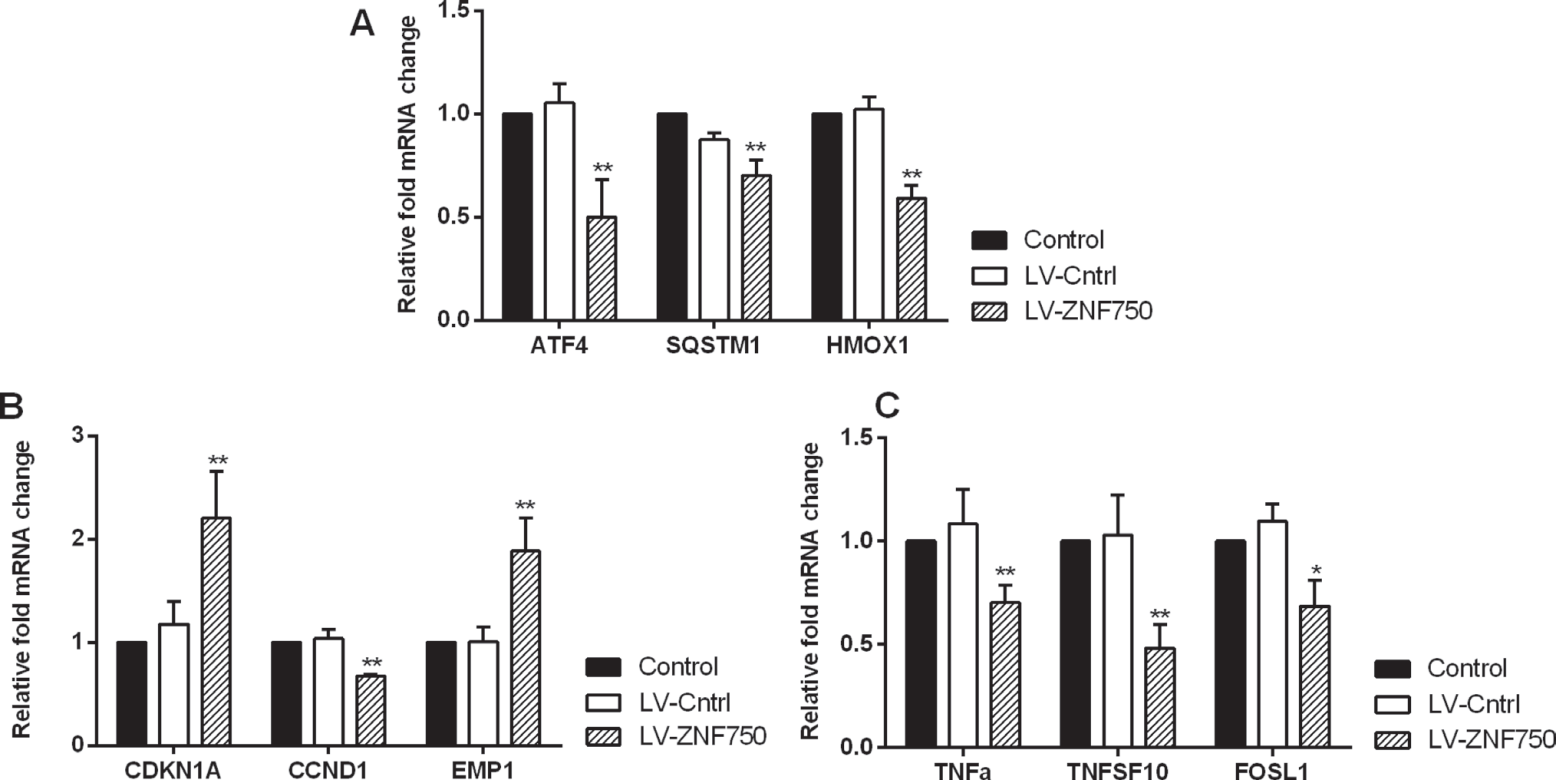
The validation for selected candidate genes by qPCR (**A–C**). Data showed the relative mRNA fold changes of candidate genes in LV-ZNF750 groups against LV-Cntrl groups. All experiments were performed in triplicate at least three times. ^*^P < 0.05, ^**^P < 0.01 *vs*. LV-Cntrl groups.

**Figure 4:**
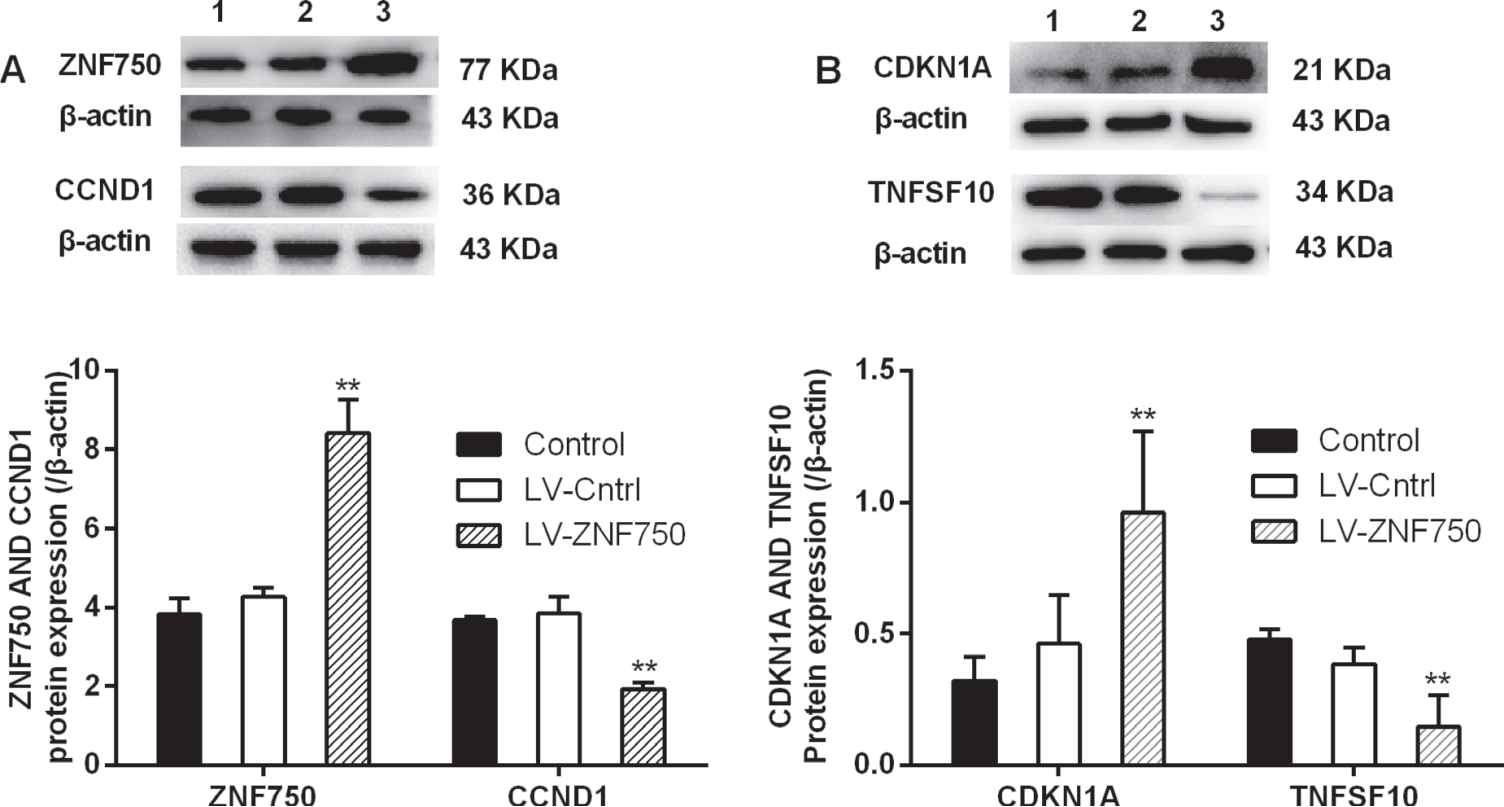
The changes of CCND1, CDKN1A and TNFSF10 protein expression upon over-expressed ZNF750 (**A** and **B**). The protein expression of selected genes was investigated by western-blot. 1: Control groups; 2: LV-Cntrl groups; 3: ZNF750 groups. ^**^P < 0.01 *vs*. LV-Cntrl groups. Each experiment was repeated at least three times.

We validate the candidate genes selection from gene profiling analyses. Our study confirmed that LV-ZNF750 group had higher CDKN1A (Cyclin-dependent kinase inhibitor 1A, p21) and EMP1 (Epithelial membrane protein 1) expression level than LV-Cntrl group. Over-expression of ZNF750 did down–regulate the ATF4 (Activating transcription factor 4), SQSTM1 (Sequestosome 1), HMOX1 (Heme oxygenase 1), CCND1, TNF-alpha (Tumor necrosis factor), TNFSF10 (Tumor necrosis factor superfamily member 10, Trail) and FOSL1 (FOS-like antigen 1) expression, approaching lower levels compared to control and LV-Cntrl groups (Figure 3, ^*^p < 0.05, ^**^p < 0.01). CDKN1A and EMP1 level upon validation did increase as preliminary results indicated in LV-ZNF750 group (^**^p < 0.01). The investigated candidate genes did not change much in LV-ZNF750 groups contrary to the array results. In consistent with the PCR array and qPCR results, CCND1, CDKN1A, TNFSF10, validation were performed by western-blot was further manifested these proteins expression changes (Figure 4, ^**^p < 0.01).

## DISCUSSION

ZNF750 is largely mutated or deleted in squamous cell carcinomas [8]. It have been reported that ZNF750 is an essential regulator of epidermal differentiation [5], and our previous studies also confirmed its function on promoting epidermal differentiation genes and repressing the epidermal progenitor genes [14]. The gene expression altered by ZNF750 may involve in the cell signal transduction process. Cancer is a typical disease exhibited abnormality cell signal transduction. In the present study, we investigated the ZNF750 protein expression changes in OSCC tumor tissues, and explored the differential gene expression of ten signal transduction pathways involved in CAL-27 cell that over-expression ZNF750 gene. Our studies revealed that ZNF750 is reduced or deleted in all detected OSCC tumor tissues. For common cell signaling analysis, ZNF750 could regulate the Hedgehog, p53, Notch, JAK/STAT, Hypoxia, WNT signaling, Oxidative Stress, NF-kappaB (NFκB), TGFβ and PPAR Signaling that related to proliferation, cell cycle, apoptosis, inflammatory, hypoxia or oxidative stress in CAL-27 cell.

The PCR array results revealed that ZNF750 down-regulated hypoxia signaling related gene ATF4, ADM (Adrenomedullin), CA9 (Carbonic anhydrase IX), EPO (Erythropoietin), SERPINE1 (Serpin peptidase inhibitor) and SLC2A1 (Solute carrier family 2) against LV-Cntrl group. Hypoxia is common characteristic of advanced tumors have been associated with poor prognosis, migration and metastasis [15]. In response to hypoxic conditions and other stresses of the tumor microenvironment, ATF4, a master transcriptional effector of the integrated stress response, is a critical mediator of cancer cell survival [16]. ATF4 expression was increased under microenvironmental stresses in tumors, leading to cancer cell adaptation and survival [17, 18]. Furthermore, Elevated ATF4 could activate antioxidant responses, including induced expression of the major antioxidant enzyme heme oxygenase 1 (HMOX-1). HMOX-1 expression was higher in human primary and metastatic tumors, increased levels of HMOX-1 ameliorated oxidative stress, cell death and promoted cell metastasis [19]. In our study, we found other oxidative stress genes, such as GSR (Glutathione reductase), TXNRD1 (Thioredoxin reductase 1) and SQSTM1 were also reduced. The SQSTM1 is frequently over-expressed in tumors and plays an important role in the regulation of tumorigenesis [20]. Consistent with above studies, our validation results found over-expression of ZNF750 could effectively reduce ATF4, HMOX-1 and SQSTM1 elevation leading to the inhibited antioxidant response activation in CAL-27 cell.

In addition to antioxidant response, inflammatory also associated with cellular transformation, cancer progression including initiation, promotion and invasion [21, 22]. In our results, the inflammatory response genes (TNF-alpha, TNFSF10, CEBPD, CSF1, ICAM1) was down-regulated in ZNF750 group. TNF-alpha (tumor necrosis factor-alpha) and TNFSF10 (Tumor necrosis factor superfamily member 10) belongs to the TNF and TNF ligand superfamily respectively. These genes encode a multifunctional pro-inflammatory cytokine. TNF-alpha had been found up-regulated in cancer patients [23]. In our study, the inhibited expression of TNF-alpha by ZNF750 may result in weak inflammatory response in CAL-27 cell. For TNFSF10, the validation for TNFSF10 did manifested descreased expression of it. Although the overwhelming majority of studies on TNFSF10 had explored the role of it as initiators of malignant cell apoptosis [24]. However, there was sporadic reports suggested that engagement of the TNFSF10 could induce cell proliferation, migratory signaling, cytokine and chemokine production [25, 26]. Therefore, the down-regulated expression of TNFSF10 by ZNF750 may inhibit cell inflammation in CAL-27 cell.

The rapid growth in tumor cells coupled with inadequate vascularization lead to shortage of oxygen (hypoxia) and nutrients. These alterations result in significant changes in gene expression [27]. The changed expression genes may involve in promoting angiogenesis, tumor growth or inhibiting cell apoptosis pathway. The PCR array result showed that EGFR (Epidermal growth factor receptor) was inhibited by ZNF750. It is well know that VEGF plays a predominant role in the increased vascularity, and VEGF signaling is partially up-regulated by EGFR expression [28]. The result also indicated the anti-angiogenesis potential of ZNF750.

The present gene expression profile revealed that the reduced pro-proliferation, and anti-apoptotic signal by ZNF750 leading to down-regulation of the genes promoting proliferation, migration or metastasis (CCND1, CCND2, GATA3, LRG1, SOCS3, FOSL1, PPARD, WISP1, BCL2, BMP2, BMP4, WNT2B, WNT5A, WNT6, HES1, HEYL, JAG1, NOTCH1, HEY1, CPT2, OLR1, SLC27A4), and genes coding the anti-apoptotic proteins (BCL2, BCL2L1). The genes involved in cell cycle arrest (CDKN1A) and tight junction (EMP1) was up-regulated by ZNF750. The well-known regulator of cell-cycle progression CCND1 and CCND2 were all decreased in our study, but the cell cycle inhibit gene CDKN1A was elevated at both mRNA and protein levels. Indicating the ZNF750 could induce the cell cycle arrest to inhibit the cancer cell proliferation. The validation of decreased FOSL1 (Fos-like antigen 1) and increased EMP1 was further define the potential anti-cancer effect of ZNF750. FOSL1 is a known proto-oncogene over-expressed in a variety of human cancers and plays important roles in oncogenesis in various malignancies [29]. Knockdown of FOSL1 in human melanoma cell lines reduced cell proliferation and migration. EMP1 is an integral tetraspan membrane protein and plays an important role in tight junction assembly [30]. Decreased expression of EMP1 was also significantly correlated with tumor invasion, metastasis, clinical stage and histological grade of patients [31]. In line with above studies, ZNF750 up-regulated the EMP1 may reduce the tumor cell invasion and metastasis.

Collectively, according to the findings of the present study, we may suggest that the reduced or deleted expression of ZNF750 in OSCC tissues linked to OSCC carcinogenesis. The signaling pathway regulated by ZNF750 support the role of ZNF750 in regulating proliferation, apoptosis, inflammation and oxidative response, unveil ZNF750 as a new the potential anti-cancer gene for OSCC. We postulate that the transcription factor ZNF750 may alters genes expression by transcriptional regulation. Therefore, further study would be necessary to understand the potential mechanism for the genes repression by ZNF750 transcriptional regulation in OSCC. It will be very helpful to elucidate the inhibited role of ZNF750 in OSCC tumor development.

## MATERIALS AND METHODS

### Specimens, cell lines and reagents

The OSCC tumors and its adjacent normal tissue from individuals were recruited from the Liaocheng people’s hospital in China between May 2004 and June 2015. The study design and protocol were reviewed and approved by the Ethics Committee of Liaocheng people’s hospital.

The 293T cells and OSCC cell line CAL-27 cells were all purchased from ATCC, pLVX-PGK-Puro lentiviral vector backbone (LV-Cntrl) and the lentiviral vector pLVX-hZNF750-PGK-Puro (LV-ZNF750) over-expression ZNF750 gene was purchased from Biowit Technologies (Shenzhen, China). The lentiviral packaging plasmids pRSV-Rev, psPax2 and VSV-G were given as a kind gift by Dr Padraig Strappe (Central Queensland University, Australian). PAHS-014Z RT^2^ Profiler™ PCR Array Human Signal Transduction PathwayFinder™ (330231) was purchased from Qiagen.

### Cell culture and treatment

The 293T packaging cells and OSCC cell line CAL27 were maintained in DMEM media supplemented with 10% FBS and 1% penicillin/streptomycin at 37°C in a humidified atmosphere containing 5% CO_2_. For signal Transduction PathwayFinder R^2^ PCR Array analysis, the CAL-27 cells growing at an exponential phase were randomly divided into two groups: LV-Cntrl groups (negative control groups, transduced with pLVX-PGK-Puro lentivirus) and LV-ZNF750 groups (transduced with pLVX-hZNF750-PGK-Puro lentivirus). For validation study, the control groups were also included.

### Lentiviral packaging and CAL-27 cells transduction

Lentiviral vector packaging and transduction was performed as we described previously with slight modification [32]. Briefly, lentiviral plasmids expressing ZNF750 gene, together with packaging plasmids pRSV-Rev, VSV-G and psPax2 were co-transfected into 70–80% confluent 293T cells with lipofectamine 2000 (Thermo Fisher Scientific, Waltham, MA, USA), according to the manufacturer’s instructions for the generation of LV-ZNF750 lentivirus. Lipofectamine 2000/DNA complexes were added into 293T cells with the addition of caffeine (final concentration of 4 mM) and sodium butyrate (final concentration of 1 mM) to achieve higher titer lentivirus [33]. Cells supernatant containing lentiviral particles was collected at 48 hr and 72 hr post-transfection, combined, and filtered through the Steriflip-HV0.45 µm PVDF Filter Unit (Millipore, Billerica, MA, USA) and concentrated by PEG-it virus precipitation solution (SBI, Palo Alto, California, USA). The CAL-27 cells were transduced with the LV-ZNF750 (multiplicity of infection, MOI = 10) in the presence of Polybrene (5 μg/mL, Sigma) after the cells reached 60–70% confluence. The cells were allowed to recover for 48 hr and puromycin (2 μg/mL, Sigma) was added into the culture medium for stable cell line selection.

### DAB immunohistochemistry

To detect the expression of ZNF750 in OSCC tumors and its adjacent normal tissues, the formalin-fixed, paraffin embedded samples were utilized for immunohistochemical analysis. Five-micron slides were cut, deparaffinized in xylene, and hydrated in decreasing concentrations of ethanol. Endogenous peroxidase was quenched with 3% hydrogen peroxide for 15 min incubation at room temperature, heated in citrate buffer (pH 6.0) in a microwave oven at 90°C for 15 min, and blocked in blocking serum. The section was incubated with primary antibody rabbit polyclonal anti-ZNF750 (1:200; Abcam, Cambridge, MA, USA) overnight at 4°C, then washed and incubated with a horseradish peroxidase (HRP) conjugated secondary antibody for 1 hr. The slides were stained using a DAB Chromogen Substrate Kit (Maxin-Bio, Co., Fuzhou, China) for 3–5 min. Images were acquired using a digital camera under the microscope (Olympus America, Melville, NY, USA).

### Real-time PCR based array analysis

An Human Signal Transduction PathwayFinder™ RT^2^ Profiler™ PCR Array (PAHS-014Z, Qiagen, Frederick, MD., USA) was used to screen a panel of 84 genes representative of ten different signal transduction pathways in CAL-27 cell under ZNF750 over-expression. Total RNA was isolated from LV-Cntrl and LV-ZNF750 group using Qiagen RNeasy Mini Kit by following manufacturer’s protocol. RNA was quantified by a Nanodrop 2000 (Gene Company Limited, Hong Kong, China) and quality assessed by visualizing 18 S and 28 S ribosomal RNA bands separated through 1% agarose with ethidium bromide staining. The first-strand cDNA was mixed with 2 × RT^2^ SYBR Green qPCR Master Mix and ddH_2_O. The qPCR was performed on an Applied Biosystems (ABI) 7500 according to the RT^2^ Profiler PCR Array instructions under the following conditions: 95°C for 10 min, then 40 cycles at 95°C for 15 sec and 60°C for 1 min. Each array contained five separate housekeeping genes (Actb, B2m, Hprt1, Ldha and Rplp1) that were used for normalization of the sample data. Microarray data was normalized against the house keeping genes by calculating the ^Δ^Ct for each gene of interest in the plate. Fold changes of gene expression, scatterplot and heatmap were analyzed and generated by using RT^2^ PCR array data analysis web portal version 3.5 (http://pcrdataanalysis.sabiosciences.com/pcr/arrayanalysis.php). Genes of ZNF750 groups that had fold changes of more than two in expression against negative control groups were considered significant. The candidate genes were chosen to be validated in an additional experiment

### Real-time PCR (qPCR)

To validate the interested genes expression changes that had fold changes of more than two, the real time PCR was performed to examine it. Total RNA from each groups was extracted with Trizol reagent (Thermo Fisher Scientific, Waltham, MA, USA), 1 μg RNA was converted to cDNA by a PrimeScript^®^ RT Kit and amplified using SYBR^®^ Premix Ex Taq™ II kit (all from Takara, Dalian, China) in a ABI 7500 Sequence Detection System. The thermal profile for qPCR was 30 s pre-incubation at 95°C for one cycle, followed by 40 cycles of 95°C for 5 s and 60°C for 34 s. Table 2 summarizes the sequences for candidate genes used in this study. The fold changes amplification for targeted genes was normalized to the housekeeping gene GAPDH by the 2-^∆∆CT^ method. Each experiment was evaluated by three PCR reactions and each experiment was repeated for at least three times.

**Table 2:** The sequences of primers

Gene	Refseq Accession #	Direction	Sequence
ATF4	NM_182810.2	Forward	CCAACAACAGCAAGGAGGAT
		Reverse	AGGTCATCTGGCATGGTTTC
CDKN1A	NM_000389.4	Forward	TGCCCAAGCTCTACCTTCC
		Reverse	CAGGTCCACATGGTCTTCCT
CCND1	NM_053056.2	Forward	GATCAAGTGTGACCCGGACT
		Reverse	TCCTCCTCTTCCTCCTCCTC
EMP1	NM_001423.2	Forward	CCAATGTCTGGTTGGTTTCC
		Reverse	GCACTGTCTTGAGGGCATCT
FOSL1	NM_005438.4	Forward	AACCGGAGGAAGGAACTGAC
		Reverse	CTTCCAGCACCAGCTCTAGG
GAPDH	NM_002046.5	Forward	TGCACCACCAACTGCTTAGC
		Reverse	GGCATGGACTGTGGTCATGAG
HMOX	NM_002133.2	Forward	AACTTTCAGAAGGGCCAGGT
		Reverse	GAAGACTGGGCTCTCCTTGTT
SQSTM1	NM_003900.4	Forward	TGCCCAGACTACGACTTGTG
		Reverse	GAGAAGCCCTCAGACAGGTG
TNF	NM_000594.3	Forward	ACCTCCTCTCTGCCATCAAG
		Reverse	CTGAGTCGGTCACCCTTCTC
TNFSF10	NM_001190942.1	Forward	CTGAAGCAGATGCAGGACAA
		Reverse	ACGGAGTTGCCACTTGACTT

### Western blot

Total protein were prepared by suspending cells in 100 μl of ice cold lysis buffer including PMSF, and centrifuged at 3000 g for 5 min before transferring the supernatant to new tubes. Protein concentration was analyzed by a BCA Protein Assay Kit (Beyotime, Jiangsu, China). Equal amounts of protein (15 µg) were subjected to 10% SDS-PAGE and were transferred to polyvinylidene difluoride (PVDF) membranes (Millipore, Bedford, MA, USA) after electrophoresis. Nonspecific bindings to the membranes were blocked with 5% skimmed milk in TBST (Tris-buffered saline-Tween 20) at room temperature for 1 hr, and followed by incubation with anti-ZNF750 (1:1000, Abcam, Cambridge, MA, USA), polyclonal antibody anti-CDKN1A (p21), anti-CCND1 (cyclin D1) and anti-TNFSF10 (1:1000 dilution, all from Bioworld Technology, St. Louis Park, MN, USA), and mouse monoclonal anti-β-actin antibody as protein loading control (1:1000 dilution, Beyotime, Jiangsu, China) overnight at 4°C. After washing with TBST for three times, the membranes were incubated with species-specific HRP-coupled secondary antibodies for 1 hr. After washing, the blots were developed using ECL western blotting kit (Beyotime, Jiangsu, China). Quantification of the protein bands was analyzed with AlphaView analysis system (ProteinSimple, Santa Clara, CA, USA). The values for CDKN1A, CCND1, and TNFSF10 proteins expression were normalized against β-actin.

### Statistical analysis

Fold changes of the transcriptional profiling of the 84 genes expression, scatterplot and heatmap were calculated and generated by using the RT^2^ PCR array data analysis web portal (version 3.5). Genes of LV-ZNF750 group with differences greater than 2-fold (*P* < 0.05) compared to LV-Cntrl group was considered significant. When comparing more than two conditions, data were analyzed by one-way ANOVA, followed by a post hoc SNK-q test. The ZNF750 protein expression in OSCC tumors and its adjacent normal tissues was evaluated using Chi-square and Fisher’s Exact Test. All data were collected and statistically analyzed using SPSS 23.0 software. Values were presented as means ± SD at least in three independent experiments. The level of statistical significance was set at *P* < 0.05.

## Abbreviations

ATF4: Activating transcription factor 4
CCND1: Cyclin D1
CDKN1A: Cyclin-dependent kinase inhibitor 1A, or p21
EMP1: Epithelial membrane protein 1
FOSL1: FOS-like antigen 1
HMOX1: Heme oxygenase 1
OSCC: oral squamous cell carcinoma
SQSTM1: Sequestosome 1
TNF-alpha: Tumor necrosis factor
TNFSF10: Tumor necrosis factor superfamily member 10, or Trail
ZNF750: Zinc-finger protein 750
